# Evaluating the Frequency of Mental Health Screening in Patients With Type 1 Diabetes Mellitus

**DOI:** 10.1192/bjo.2026.11835

**Published:** 2026-06-30

**Authors:** Friederike Schaper, Kamal Abouglila

**Affiliations:** 1 NHSE Education North East, Newcastle upon Tyne, United Kingdom; 2 County Durham and Darlington NHS Foundation Trust, Durham, United Kingdom

## Abstract

**Aims:**

Type 1 diabetes mellitus (T1DM), an autoimmune disease resulting in insulin deficiency and hyperglycaemia, is associated with a considerable burden of psychiatric comorbidities, particularly mood, anxiety and eating disorders. As such, regular mental health screening for T1DM patients has been recommended to allow earlier intervention. This audit aimed to investigate whether T1DM patients in our trust (County Durham and Darlington Foundation Trust, CDDFT) had received psychological screening at time of diagnosis and within the past year.

**Methods:**

A questionnaire consisting of six clinician-designed self-report items assessing patient recall of mental health screening at diagnosis and within the past year was distributed at regular T1DM clinic appointments at University Hospital of North Durham and Chester-Le-Street Community Hospital by clinicians involved in T1DM care. All patients with T1DM under regular follow-up within these centres were eligible; there were no defined exclusion criteria regarding age or other demographic factors.

**Results:**

A total of 23 responses were received, of which four were excluded due to inconsistencies. Of the 19 included responses, 32% (n=6) stated that they had received mental health screening at diagnosis. When analysed further by age, it was found that none of the seven individuals diagnosed before the age of 18 had received screening at diagnosis. Overall, only 26% (n=5) of respondents indicated that they had received mental health screening as part of their T1DM care within the past year. Interestingly, only 21% (n=4) of respondents stated that they would appreciate more frequent mental health screening, while 32% (n=6) did not answer this question, with one individual responding “Only if it meant extra support”.

**Conclusion:**

Given the high impact of a T1DM diagnosis on psychological wellbeing, and the strong link to various psychiatric conditions, the reported low levels of mental health screening are concerning. At present, mental health screening in T1DM care at CDDFT is clinician-dependent with no standardised process. It is interesting to consider whether factors such as a lack of awareness, time pressure within appointments, or a perceived ‘helplessness’ on the part of the clinician in terms of organising appropriate mental health follow-up, are resulting in the low rates of screening identified, although limitations including risk of recall bias and small sample size are important to take into account. Going forward, we aim to present this data to clinicians involved with T1DM care within the trust and recommend starting all consultations with a short, validated screening tool such as the Diabetes Distress Scale.

